# Right-sided diaphragmatic rupture after blunt trauma. An unusual entity

**DOI:** 10.1186/1749-7922-6-3

**Published:** 2011-01-18

**Authors:** Ramon Vilallonga, Vicente Pastor, Laura Alvarez, Ramon Charco, Manel Armengol, Salvador Navarro

**Affiliations:** 1 General Surgery Department. Endocrine, bariatric and metabolic Unit. Universitary Hospital Vall d'Hebron. Autonomous University of Barcelona. Spain; 2 General Surgery Department. Universitary Hospital Vall d'Hebron. Autonomous University of Barcelona. Spain; 3 HBP Surgery and Transplants Department. Universitary Hospital Vall d'Hebron. Autonomous University of Barcelona. Spain; 4 HBP Surgery and Transplants Department. Universitary Hospital Vall d'Hebron. Autonomous University of Barcelona. Spain; 5 General Surgery Department. Universitary Hospital Vall d'Hebron. Autonomous University of Barcelona. Spain; 6 General Surgery Department. Universitary Hospital Parc Tauli. Autonomous University of Barcelona. Spain

## Abstract

Traumatic injuries of the diaphragm remain an entity of difficult diagnosis despite having been recognised early in the history of surgery, especially when it comes to blunt trauma and injuries of the right diaphragm. We report the case of a patient with blunt trauma with right diaphragmatic rupture that required urgent surgical treatment for hepatothorax and iatrogenic severe liver injury. Blunt trauma can cause substantial diaphragmatic rupture. It must have a high index of suspicion for diaphragmatic injury in patients, victims of vehicle collisions, mainly if they have suffered frontal impacts and/or side precipitates in patients with severe thoracoabdominal trauma. The diagnosis can be performed clinically and confirmation should be radiological. The general measures for the management of multiple trauma patients must be applied. Surgery at the time of diagnosis should restore continuity.

## Introduction

Traumatic injuries of the diaphragm remain an entity of difficult diagnosis despite having been recognised early in the history of surgery. Sennertus, in 1541, performed an autopsy in one patient who had died from herniation and strangulation of the colon through a diaphragmatic gap secondary to a gunshot wound received seven months earlier [1]. However, these cases remain rare, and difficult to diagnose and care for. This has highlighted some of the aspects related to these lesions, especially when they are caused by blunt trauma and injuries of the right diaphragm [1,2].

## Case report

**Figure 1:**
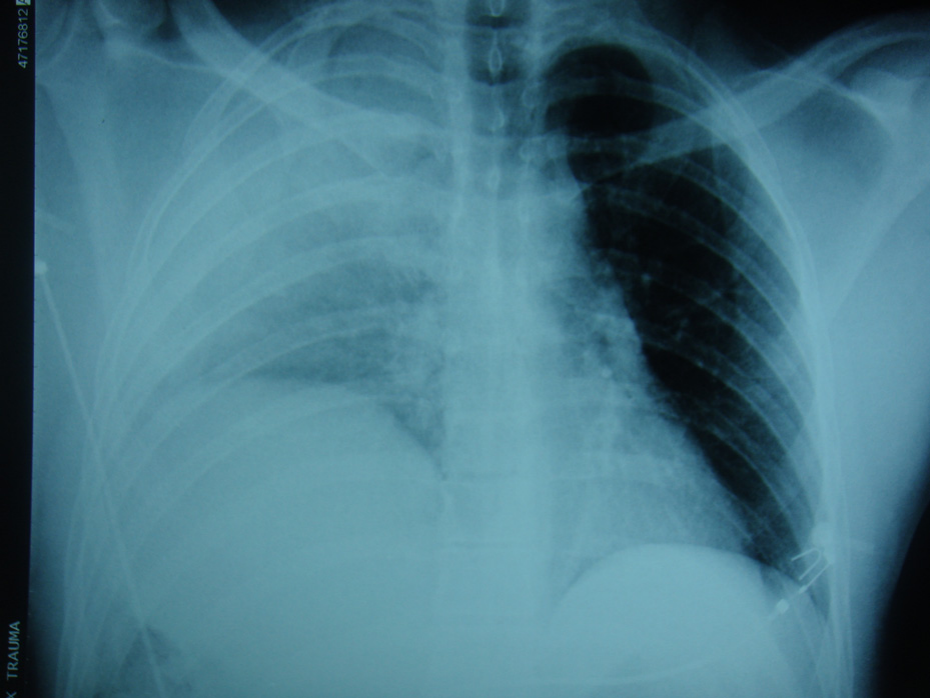
**Chest radiograph of the patient showing an elevated right hemidiaphragm**.

**Figure 2:**
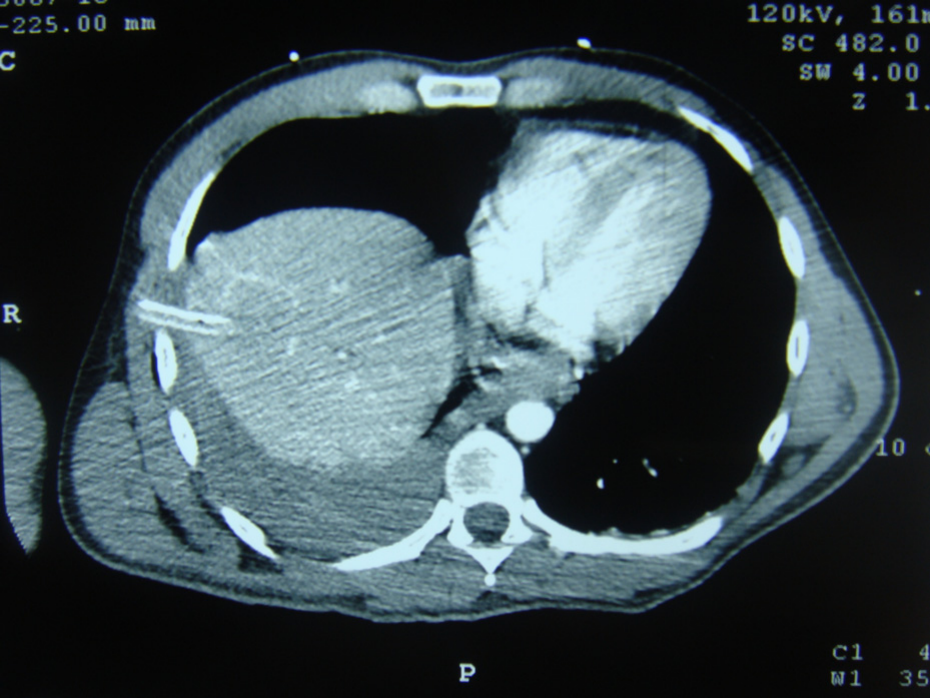
**CT scan of the patient where hepatothorax is displayed with the drain inside**.

We report the case of a man of 36 years of age, thrown from a height of 12 meters and was referred to our centre. The patient arrived conscious and oriented, and we began manoeuvring the management of the patient with multiple injuries according to the guidelines of the ATLS (Advanced Trauma Life Support) recommended by the American College of Surgeons. The patient had an unstable pelvic fracture (type B2) with hemodynamic instability and respiratory failure. Patient's Injury Severity Score (ISS) was 38. Pelvis and chest X-rays were performed which confirmed the pelvic fracture and pathological elevation of the right hemidiaphragm was observed (Figure 1). We proceeded to stabilise the pelvic fracture and replace fluids, improving hemodynamic status. The patient continued with respiratory failure. For this reason, a chest tube was placed and Computerised Tomography (CT) was performed (Figure 2), showing a ruptured right hemidiaphragm, including chest drain in the right hepatic lobe and occupation of the lesser sac by blood. The patient underwent surgery, finding a right hemidiaphragm transverse rupture with a hepatothorax and an intrahepatic thoracic tube. We performed the suture of the diaphragm and liver packing, moved the patient to the intensive care unit, and after 48 hours, the liver packing was removed without problems. The patient evolved favourably.

## Discussion

Currently, traumatic injuries of the diaphragm remain uncommon, and it is difficult to establish a global impact, but by autopsy studies, the incidence of these injuries range between 5.2% and 17% [3]. If we focus on patients with blunt trauma, we find that traumatic injuries of the diaphragm represent only 0.8% to 1.6% of the total lesions observed in these patients [4]. However, when we talk about open trauma, these injuries may represent up to 10% -15% of cases [3,5,6].

Road traffic collisions or lateral intrusions into the vehicle are the most frequent causes of diaphragm rupture [1,4,6,7]. Direct impacts depress the side of the rib cage, and can cause a tear in the diaphragm rib attachments, and even the transverse rupture of the diaphragm [8]. Also, serious slowdown pinching leads to a multiplication by ten times or more to the intra-abdominal pressure, especially if the patient holds his/her breath and contracts the abdominal wall at the time of impact, causing a muscle injury [2].

Classically, there has been a predominance of lesions of the left hemidiaphragm, with a ratio of 25:1. However, most modern series balance this data and show that right hemidiaphragm injuries can represent almost 35% of all diaphragm injuries [9]. This pattern may explain why the liver develops a protective cushioning pressure, although some authors believe that right hemidiaphragm injuries are associated with increased mortality so would be undiagnosed, and for this reason would be found in equal proportion at autopsy [4,6,8].

Many authors have reviewed blunt diaphragmatic trauma over a period in their institutions. We do report the major reviewed series to our knowledge in which the do a specific mention to the blunt abdominal trauma associated with diaphragmatic rupture (Table 1).

**Table 1:** Major series reporting cases in the literature of blunt diaphragmatic rupture.

Author	Number of cases	Trauma type	Location	Associated injuries	ISS*	Management	Mortality
Chughtai T et al. [9]	208 (1986-2003)	Blunt: 208	Right: 135 Left: 47 Bilateral: 4	Abdomen: liver (63,5%), spleen (52,9%), small bowel mesentery (46,2%)... Chest: Rib fracture (75,5%), pulmonary contusion (63,0%), hemothorax (40,4%), hemopneumothorax (22,1%)...	Mean ISS 38.0	93,3% laparotomy 1,4% thoracotomy	60 † within 28 days. Head injury: 25% Intra-abdominal bleeding: 23,2%

Ozpolat B et al. [7]	41 (1996-2007)	Blunt: 20 Penetrating: 21	Right: 12 Left: 28 Bilateral: 1	30 (73%): hemothorax, pneumothorax, liver and rib fractures	Not mentioned.	85% operated before 24 h	6 † (14,6%)

Lunca S et al. [12]	61 (1992-2003)	Blunt: 15 Penetrating: 46	Right: 15 Left: 45 Bilateral: 1	27 hemorrhagic shock	ISS = 24 (6-75)	100% operated before 12 h	9 † 15 complications

Cubukçu A et al. [13]	21 (1995-1998)	Blunt: 9 Penetrating: 12	Right: 12 Left: 9	20 patients with concomitants injuries (Liver in 10 patients) 7 patients with signs or symptoms related to diaphragmatic rupture	Not mentioned.	100% operated before 24 h	3 †

Dajee A et al. [14]	48 (1973-1978)	Blunt: 8 Penetrating: 40	Right: + Left: +++ Bilateral: 1	Intra-abdominal injuries involved the spleen, liver, stomach and colon. 8 patients herniations of intra-abdominal contents.	Not mentioned	100% laparotomy. No use of mesh.	3 † (6%)

Tan KK et al. [16]	14 (2002-2008)	Blunt: 14	Right: 5 Left: 9	8 Splenic laceration, 5 hemothorax and lung injuries, 4 long bone fracture, 4 pelvic fracture, 3 liver laceration, 3 colonic laceration, 3 injury major vessels, 2 kidney laceration, 2 small bowel laceration, 1 gastric perforation.	Median GCS: 14 (3-15) Median ISS: 41 (14-66).	85,7% laparotomy and repair 14,3% surgical intensive care unit.	5 † (33%) Extensive injuries

Matsevych OY. [19]	12 (4 years)	Blunt: 12	Right: 6 Left: 2 Bilateral: 1	100% associated injuries: 5 hemothorax, 4 head injuries, 3 extremity fracture, 3 pelvic fracture, 3 liver laceration, 3 retroperitoneal hematoma.	Not mentioned.	100% laparotomy. 1 patient thoracotomy.	3 † (25%) (Hypovolemic shck, 1 brain injury, 1 cardiac failure)

Bergeron E et al. [20]	160 (April 1, 1984, to March 31, 1999)	Blunt: 160	Right: 31 Left: 126 Bilateral: 3	Abdomen: liver (47%), spleen (50%), small bowel mesentery (38%)... Chest: Rib fracture (31%), pevi (41%), other orthopedic (50%).	ISS = 26.9 (+-11.5)	100% operated between 60 minutes and 21.8 days after injury. 4 had repair of diaphragmatic rupture at a second laparotomy.	14,4%

Brasel KJ et al. [21]	32 (January 1987 through May 1994)	Blunt: 32	Right: 7 Left: 25 Bilateral: 0	Abdomen: liver (47%), spleen (50%), small bowel mesentery (38%)... Chest: Rib fracture (31%), pevi (41%), other orthopedic (50%).	ISS= 32	100% laparotmomy. Sunning suture all patients and 1 patient polypropylene mesh repait.	22,0%

Shapiro MJ et al. [22]	20 (5 years period)	Blunt: 20	Right: 7 Left: 14 Bilateral: 0	Shock 16/20; hemo/pneumothorax 15/20; cerebral injury (12/20); puhnonary contusion 9/20; chest wall contusion 8/20; hepatic injury 8/20; splenic injury 8/20	36 (11-59)	Not mentioned	25,0%

Montresor E et al. [23]	17 (1970 to 1995)	Blunt: 17	Right: 7 Left: 14 Bilateral: 0	52.6% presented at operation with intrathoracic visceral herniation.	Not mentioned.	8 laparotomy. 7 laparotomy and thoracotomy. 4 thoracotomy	15,6%

Esme H et al. [24]	14 (January 2000 and June 2005)	Blunt: 11 Penetrating: 3	Right: 4 Left: 10	Multiple associated injuries were observed in 12 patients (85%)	Not mentioned.	100% laparotomy.	Overall: 7%

Athanassiadi K et al. [25]	41 (1988 to 1997)	Blunt: 41	Right: 15 Left: 24 Bilateral: 2	In 34 patients (94%) involving: spleen (n = 18), rib fractures (n = 17), liver (n = 14), lung (n = 11), bowel (n = 7), kidney (n = 5) and other fractures (n = 21)	Not mentioned.	22 laparotomy 10 thoracotomy 4 laparo-thoracotomy	16.6% (6/36)

Gwely NN. [26]	44 (1998 and 2007)	Blunt: 44	Right: 12 Left: 30 Bilateral: 2		Not mentioned.	31 thoracotomy in 4 laparotomy 3 thoracolaparotomy	13.2% (5/38)

Yalçinkaya I et al. [27]	26 (1996-2005)	Blunt: 26	Right: 8 Left: 18	Multiple associated injuries were observed in patients (96%). Thorax herniation of organs (45%).	Not mentioned.	15 thoracotomy 7 laparotomy 4 thoraco-laparotomy	3 † (11.5%)

* Injury Severity Score

The clinical presentation is defined by the overall assessment of the patient with multiple injuries. The injury must be suspected when any hemidiaphragm is not seen or not in the correct position in any chest radiograph [15]. The specific signs of diaphragmatic injury on plain radiographs are a marked elevation of the hemidiaphragm, an intrathoracic herniation of abdominal viscera, the "collar sign", demonstration of a nasogastric tube tip above the diaphragm [19]. Also, in the context of high-energy trauma, when combined with a head injury and pelvic fracture, diaphragmatic trauma should be suspected [7]. The diagnosis is based largely on clinical suspicion and a compatible chest radiograph or CT scan [10]. The biggest change in recent years in managing blunt diafragmatic trauma has been the use of high-resolution multislice CT angiography of the abdomen and chest. This is now a routine test performed in most blunt trauma patients. Ultrasound can also be diagnostic in patients with DR, especially if focused abdominal sonography for trauma (FAST) can be extended above the diaphragm looking for a hemothorax and assessing the diaphragmatic motions (using m-mode if possible). It adds little time to the examination but allows the operator to observe absent diaphragmatic movements, herniation of viscera, or flaps of ruptured diaphragm [19]. However, in the absence of a hernia, it may be difficult to identify traumatic diaphragmatic injury by conventional imaging. Blunt diaphragmatic rupture is often missed during initial patient evaluation. The initial chest radiograph can be negative and a repeat chest radiograph may be necessary. Other diagnostic modalities or even surgical exploration may be required to definitively exclude blunt diaphragmatic rupture. A midline laparotomy is the advocated approach for repair of acute diaphragmatic trauma because it offers the possibility of diagnosing and repairing frequently associated intra-abdominal injuries [11].

Closed diaphragmatic injuries should be treated as soon as possible. Special attention should be given to the placement of thoracic drainage tubes, especially if the radiograph is suspicious [3].

Midline laparotomy is the recommended approach because it allows for an exploration of the entire abdominal cavity [1,2,4,6,7]. Routine surgical repair of any diaphragmatic defect is accomplished by interrupted or continuous nonabsorbable sutures and placement of chest tube(s) in the affected thoracic cavity. In hemodynamically stable patients with penetrating left thoracoabdominal trauma, the incidence of injury to the diaphragm is very high, and thoracoscopy or laparoscopy is recommended for the diagnosis and repair of a missed diaphragmatic injury. Laparoscopy or video-assisted thoracoscopic surgery (VATS) can be used in hemodynamically stable patients. VATS has greater accuracy (sensitivity and specificity close to 100%) and helps to avoid the risk of tension pneumothorax [19]. However, we feel that VATS is best reserved for stable patients when intraabdominal and contralateral diaphragmatic injuries are excluded.

Grimes, in 1974, described the three phases of the rupture of the diaphragm: an initial acute phase, at the time of the injury to the diaphragm; [17] a delayed phase associated with transient herniation of the viscera, thus accounting for absent or intermittent non-specific symptoms; and the obstruction phase involving the complication of a long-standing herniation, manifesting as obstruction, strangulation and posterior rupture [18]. The typical organs that herniate into the thoracic cavity include the stomach, spleen, colon, small bowel and liver, Repair with non-absorbable simple sutures is adequate in most cases, and the use of mesh should be reserved for chronic and large defects. Thus, all surgeons must be vigilant during any exploratory laparotomy to exclude any associated diaphragmatic injury.

Mortality strictly related to diaphragmatic rupture is minimal, and is usually caused by the associated injuries. The most common causes of death reported in the literature are shock, multiple organ failure and head injuries [9]. Outcomes of acute diaphragmatic hernia repair are largely dictated by the severity of concomitant injuries, with the Injury Severity Score being the most widely recognised predictor of mortality. Delayed diagnosis may increase mortality by up to 30% [8]. The rate of initially missed diaphragmatic ruptures or injuries in nonoperatively managed patients, therefore, ranges from 12 to 60% [3]. Blunt diafragmatic rupture can easily be missed in the absence of other indications for prompt surgery, where a thorough examination of both hemidiaphragms is mandatory. A high index of suspicion combined with repeated and selective radiologic evaluation is necessary for early diagnosis. Acute diaphragmatic hernia is a result of diaphragmatic injury that accompanies severe blunt or penetrating thoracoabdominal trauma. It is frequently diagnosed early on the trauma by chest radiograph or CT scan of the chest. Non-adverted diaphragmatic injury resulting from the chronic phase of a diaphragmatic hernia will probably require surgery to repair the defect.

## Conclusions

Blunt diaphragmatic rupture can lead to important morbidity and mortality. It is a rare condition, usually masked by multiple associated injuries, which can aggravate the condition of patients. Therefore, there should be a high index of suspicion for diaphragmatic injury in those patients who are victims of vehicle collisions, especially if they have suffered frontal and/or lateral impacts, which have resulted in severe thoracoabdominal trauma. The diagnosis can be made clinically and radiologically. The general measures for the management of multiple trauma patients must be applied. Surgery at the time of diagnosis should restore continuity.

## Competing interests

Dr. Ramon Vilallonga is president of the Dr. Vilallonga Foundation. The rest of authors, declare that they have no competing interests.

## Authors' contributions

VR has take care of the patient and has draft the manuscript. PV, AL, CR helped to the clinical assessment and draft of the manuscript. CR, AM and NS have been involved in drafting the manuscript or revising it critically for important intellectual content. All authors read and approved the final manuscript.
